# Urban agriculture in walkable neighborhoods bore fruit for health and food system resilience during the COVID-19 pandemic

**DOI:** 10.1038/s42949-023-00083-3

**Published:** 2023-02-01

**Authors:** Akiko Iida, Takahiro Yamazaki, Kimihiro Hino, Makoto Yokohari

**Affiliations:** 1 Department of Urban Engineering, Graduate School of Engineering, The University of Tokyo, Tokyo, 1138656 Japan; 2 Department of Environmental Design, Kobe Design University, Hyogo, 6512196 Japan

**Keywords:** Urban ecology, Quality of life, Civil engineering, Environmental studies, Agriculture

## Abstract

Urban agriculture is the key to creating healthy cities and developing resilient urban food systems in uncertain times. However, relevant empirical evidence is limited. This study quantitatively verified the association of access to local food through urban agriculture with subjective well-being, physical activity, and food security concerns of neighborhood communities in the context of the COVID-19 pandemic. The target was Tokyo, Japan, where small-scale local food systems are widespread in walkable neighborhoods. We found that diversity in local food access, ranging from self-cultivation to direct-to-consumer sales, was significantly associated with health and food security variables. In particular, the use of allotment farms was more strongly associated with subjective well-being than the use of urban parks, and it was more strongly associated with the mitigation of food security concerns than the use of food retailers. These findings provide robust evidence for the effectiveness of integrating urban agriculture into walkable neighborhoods.

## Introduction

Food issues in urban areas, where more than half the world’s population has lived since the mid-2000s, are becoming a crucial global challenge^1^. In this context, local food production through urban agriculture is attracting attention as a viable alternative to conventional industrial agriculture^2,3^. This is due to the multidimensional benefits of urban agriculture, which contribute to urban sustainability and resilience. Such benefits include the improvement of food security and food system resilience, mitigation of climate risks, conservation of biodiversity, development of social capital, and promotion of the health and well-being of urban residents^4–6^. In addition, urban agriculture may bring external benefits to the global environment by reducing the carbon footprint of food production and distribution processes and by changing food consumption for greener diets^7–10^. Therefore, urban agriculture has become an emerging topic of urban planning and urban food policies^11,12^.

In addition, the concept of walkable neighborhoods where people can access basic urban amenities within walkable distances (e.g., the 15-min city model) is gaining global traction^13–15^. Walkable neighborhoods are expected to contribute to climate change mitigation by limiting vehicle travel and reducing carbon emissions; furthermore, walking and cycling promote health and well-being^13–16^. A study that identified measurable attributes of walkable neighborhoods showed that in addition to residential density, block size, and street connectivity, the food environment, such as the distance to food retail facilities, is a critical component^17^.

Against this backdrop, the coronavirus disease 2019 (COVID-19) pandemic has acted as a trigger. In view of increased food insecurity, it has raised global awareness of the importance of strengthening urban agriculture to develop more resilient urban food systems and create healthier cities^18,19^. The walkable neighborhood concept has also earned worldwide attention, as urban residents have had to spend more time in their homes and neighborhoods owing to infection control restrictions^20^.

However, the two concepts of urban agriculture and walkable neighborhoods are not fully integrated. Food access, especially fresh food access, is a basic need that should be satisfied at the neighborhood level^13,17^. There has been much research on food deserts, i.e., geographical areas that lack fresh food access and have high food insecurity, particularly focusing on low-income communities^21,22^. However, such studies mainly focus on access to food retailers, failing to explore local food production through urban agriculture^23^. Furthermore, while some studies have theoretically explored the potential of local food production by urban communities as a means of improving food access and reducing food insecurity, these studies have generally been qualitative. Thus, quantitative evidence is limited^24–26^.

In addition, recent studies suggest that food system resilience is important for food security, which is the capacity of food systems to adapt to changes in external and internal disruptions with sufficient, appropriate, and accessible food for all^10,27^. During the COVID-19 pandemic, many opinions and perspectives were published, describing the importance of urban agriculture for food security and food system resilience in times of uncertainty^18,19^. A few studies explored the characteristics of gardeners in North America^28–30^ and the perceptions and values of gardening^31^. However, to the best of our knowledge, no research that empirically investigates food system resilience driven by urban agriculture has been undertaken.

The other potential advantage of urban agriculture is the health benefit urban communities are afforded by engaging in farming and gardening. Recent epidemiological studies have provided significant evidence that the existence of urban green spaces and the experience of nature they offer are associated with cognitive development, mental health, physical health, and well-being^32–35^, even during the COVID-19 pandemic^36,37^. Thus, urban nature has been recognized as an effective measure for improving public health. Urban farming and gardening, which are types of nature experiences, have also been found to be associated with mental and physical health^38–40^. However, few studies consider the differences between various types of urban green spaces^33^; the experience of nature through urban farming and gardening might differ from such an experience in other urban green spaces, such as urban parks.

To fill these knowledge gaps, we conducted a case study in Tokyo, where urban agriculture is widely practiced in densely populated urban neighborhoods and people can access local fresh foods on foot or by bicycle within walkable neighborhoods. We aimed to empirically verify the association of access to local food through urban agriculture with subjective well-being, physical activity, and food security concerns in the context of the COVID-19 pandemic. Then we discussed the benefits of the integration of urban agriculture into walkable neighborhoods from health and food system resilience perspectives. We performed logistic regression analyses using a cross-sectional dataset of 3,135 adults collected by an online questionnaire survey between June 4 and 8, 2020, to investigate the following three specific objectives:

1. To examine the association of three types of access to local food (allotment farms, home gardens, and farm stands) with subjective well-being, physical activity, and food security concerns during the state of emergency and in the future.
2. To identify characteristics of access to local food in comparison with access to other urban green spaces (small parks, large parks, and greenways) and to other food purchasing sites (grocery stores, convenience stores, and co-op deliveries).
3. To investigate the characteristics of those who accessed local food during the pandemic, particularly focusing on work style and income highly affected by the pandemic.

**Fig. 1:**
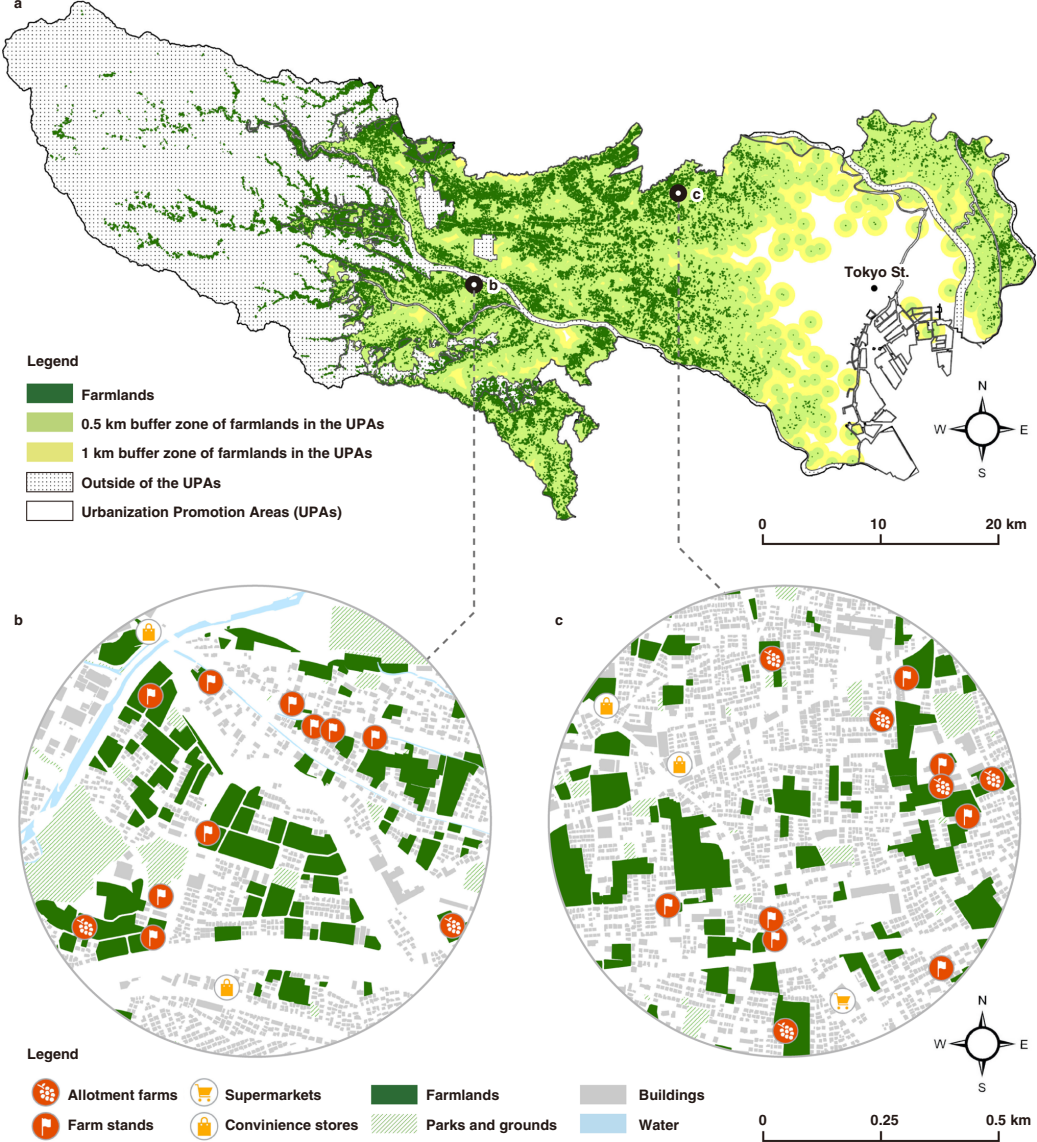
Urban farmlands in Tokyo. **a** Farmlands and their buffer zones of 0.5 and 1 km radiuses in the urbanization promotion areas (UPAs). **b**, **c** Close-up maps of the 0.5 km radius areas indicated by the black line circles on Map **a**. These maps show examples of residential neighborhoods with a mixture of agricultural land in Hino City (**b**) and Nerima Ward (**c**). Residents can access allotment farms and farm stands within walkable distances (Map **a** excludes the islands in Tokyo).

While urban agriculture in cities includes both intraurban and peri-urban agriculture^41^, this study focuses on intraurban areas owing to the following sociogeographical characteristics of Tokyo: The suburbs of Japanese cities have a unique horizontal distribution structure for urban‒rural mixed landscapes^42^ (Fig. 1). This phenomenon directly contrasts with the global trend of the vertical structure of urban agriculture, such as farming in buildings or on rooftops with advanced technologies. This structural feature of Japanese cities has created a unique local food system. Urban residents can buy and/or produce local fresh foods near their homes. The intraurban areas in Tokyo, known as urbanization promotion areas (UPAs), included 43.1 km^2^ of urban farmlands in 2016‒2017, and 71 and 84% of these areas provide access to residents within a 0.5 and a 1 km radius, respectively (Fig. 1 and Supplementary Table 1). There is no universal definition of the local food distance between food producers and consumers. For example, it is defined as under 400 miles (~644 km) in the United States, 50 km in Canada, and 20–100 km in the European Union^43^. In contrast, in Japanese major cities, including Tokyo, it is a few kilometers or less and is thus accessible on foot or by bicycle. The local food system and its foodshed are very small, decentralized, and community-based.

**Fig. 2:**
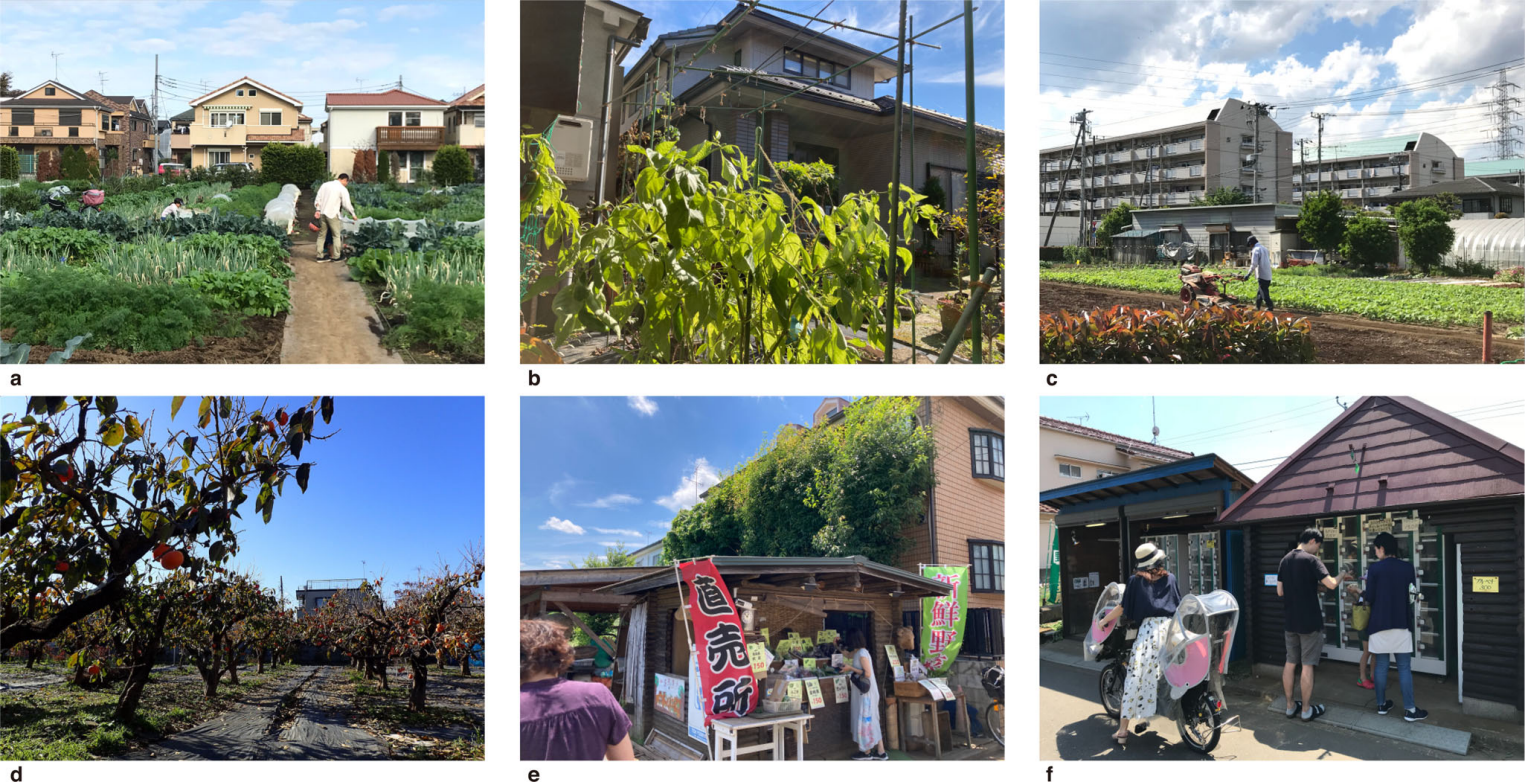
Key places of urban agriculture in Japanese cities. **a** Allotment farms. **b** Home gardens. **c**, **d** Commercial urban farmlands owned by professional farmers in residential neighborhoods. A vegetable farm (**c**) and a fruit farm (**d**). **e**, **f** Farm stands called “*choku bai jyo*”: an open-type farm stand (**e**) and a vending machine-type farm stand (**f**).

Globally, there are various types of urban agriculture, including horticulture, animal husbandry, aquaculture, and arboriculture^44^. All of these types can be found in Tokyo, but we focused on the three most common types of horticultural activities: allotment farming, home gardening, and commercial farming (Fig. 2 and Supplementary Note 1). Allotment farms are divided into lots of ~10 to 30 m^2^ and rented out to individuals or groups^40^. Home gardens vary in size, but in Tokyo, where land prices are high, the size of home gardens is generally smaller than that of allotment lots. Regarding commercial farming, most farmers are small-scale, with less than 10,000 m^2^ of farmlands, most of which are operated as family businesses. Farmers produce mainly vegetables and fruits and ship their products in various ways, including to wholesale markets. However, in Tokyo, direct-to-consumer sales, especially at farm stands known as “*choku bai jyo*,” are more popular. Thus, we focused on direct-to-consumer sales at farm stands for commercial farming. We consider Tokyo a referential case that can provide robust evidence for urban policies to promote urban agriculture.

## Results

### Subjective well-being and physical activity

Binomial logistic regression analyses were conducted to assess the association of subjective well-being with access to local food (Model 1a) and to green spaces (Model 1b), as well as the association of physical activity with access to local food (Model 2a) and to green spaces (Model 2b). We developed additional models to compare each of the three types of access to local food to access to green spaces for subjective well-being (Model 1c–1e) and for physical activity (Model 2c–2e) (Supplementary Fig. 1). All models were adjusted for sociodemographic control variables.

**Fig. 3:**
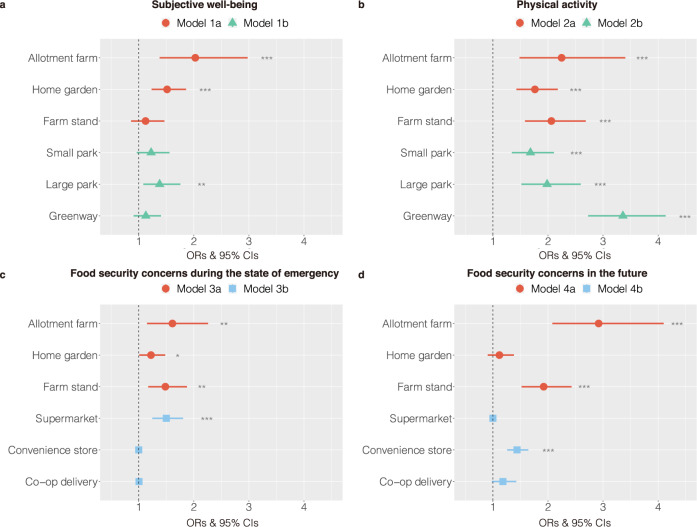
The associations between local food access and health and food security during the COVID-19 pandemic. **a**, **b** The associations of access to local food and other urban green spaces with subjective well-being (**a**) and physical activity (**b**). **c**, **d** The associations of access to local food and other food purchasing places with food security concerns during the state of emergency (**c**) and in the future (**d**). The circle (red), triangle (green), and square (blue) symbols indicate the ORs. The vertical dashed line is the reference line and is set to one. The error bars represent the 95% CIs. Predictors are interpreted to be significant if their 95% CIs do not cross one line. The significant results are shown as follows: *p < 0.05, **p < 0.01, and ***p < 0.001.

**Fig. 4:**
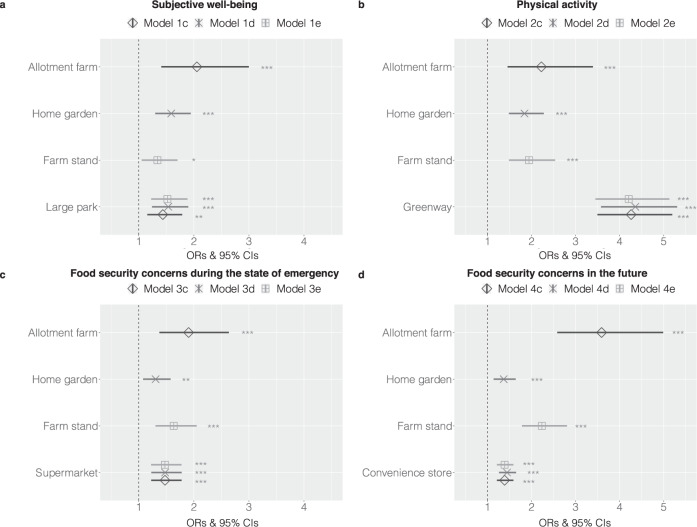
The comparisons between access to local food, other urban green spaces, and other food purchasing places. **a**–**d** The associations of subjective well-being with each type of local food access and large parks (**a**), physical activity with each type of local food access and greenways (**b**), food security concerns during the state of emergency with each type of local food access and supermarkets (**c**), and food security concerns in the future with each type of local food access and convenience stores (**d**). The diamond (◊), cross (x), and square (□) symbols indicate the ORs. The vertical dashed line is the reference line and is set to one. The error bars represent the 95% CIs. Predictors are interpreted to be significant if their 95% CIs do not cross one line. The significant results are shown as follows: *p < 0.05, **p < 0.01, and ***p < 0.001.

Regarding subjective well-being, users of allotment farms and home gardens had significantly higher scores than those who did not use them (Fig. 3a and Supplementary Table 4). No significant differences for users of farm stands were found. Among urban green spaces, only users of large parks had significantly higher scores than those who did not use them, but small parks or greenways indicated no significance. In the analysis comparing access to local food and green space, allotment farms were more strongly associated with subjective well-being (odds Ratio (OR): 2.06, 95% confidence interval (CI): 1.41, 3.00, *p* < 0.001) than large parks (OR: 1.44, 95% CI: 1.16, 1.79, *p* = 0.001) (Fig. 4a and Supplementary Table 5). Home gardens (OR: 1.59, 95% CI: 1.30, 1.95, *p* < 0.001) also had a slightly stronger association than large parks (OR: 1.54, 95% CI: 1.24, 1.90, *p* < 0.001).

Regarding physical activity, users of allotment farms, home gardens, or farm stands had significantly greater physical activity than those who did not use them (Fig. 3b and Supplementary Table 4). Among them, allotment farms (OR: 1.61, 95% CI: 1.15, 2.26, *p* < 0.01) were more strongly associated with physical activity than home gardens (OR: 1.22, 95% CI: 1.01, 1.48, *p* < 0.05) and farm stands (OR: 1.48, 95% CI: 1.17, 1.88, *p* < 0.01). In addition, users of small parks, large parks, or greenways had significantly greater physical activity. Among them, greenways (OR: 3.36, 95% CI: 2.73, 4.14, *p* < 0.001) had the strongest association. In the analysis comparing access to local food and green space, greenways exceeded allotment farms, home gardens, and farm stands (Fig. 4b and Supplementary Table 5).

### Food security concerns (anxiety about the availability of fresh food)

Ordinal logistic regression analyses were performed to determine the association of food security concerns during the state of emergency with access to local food (Model 3a) and to food retailers (Model 4a), as well as the association of food security concerns in the future with access to local food (Model 3b) and to food retailers (Model 4b). Further, we developed additional models to compare each of the three types of access to local food to access to food retailers during the emergency (Model 3c–3e) and in the future (Model 4c–4e) (Supplementary Fig. 1). All models were adjusted for sociodemographic control variables.

Those who used either allotment farms or farm stands had significantly lower anxiety about the availability of fresh food both during the state of emergency and in the future than those who did not (Fig. 3c, d and Supplementary Table 6). Notably, the OR of allotment farms (OR: 2.92, CI: 2.08, 4.10, *p* < 0.001) was especially high for anxiety regarding the future. Users of home gardens (OR: 1.22, CI: 1.01, 1.48, *p* = 0.04) had significantly lower anxiety about the availability of fresh food during the state of emergency, but regarding the future, there were no significant differences. Among other purchasing sites, supermarket users had significantly lower anxiety during the state of emergency, and convenience store users had significantly lower anxiety regarding the future, but no significant differences for users of co-op deliveries were found.

In the analysis comparing access to local food and other food purchasing sites, allotment farms and farm stands had higher ORs than supermarkets for anxiety about the availability of fresh food during the state of emergency and had higher ORs than convenience stores for anxiety regarding the future (Fig. 4c, d and Supplementary Table 7). In contrast, home gardens had significantly positive ORs for anxiety about the availability of fresh food during the state of emergency and in the future, the strengths of which were slightly lower than those of supermarkets and convenience stores.

### People who access local food

Binomial logistic regression analyses were conducted to identify the characteristics of those who accessed each of the three types of local food: allotment farms (Model 5a), home gardens (Model 5b), and farm stands (Model 5c). The explanatory variables of these models comprised sociodemographic characteristics.

**Fig. 5:**
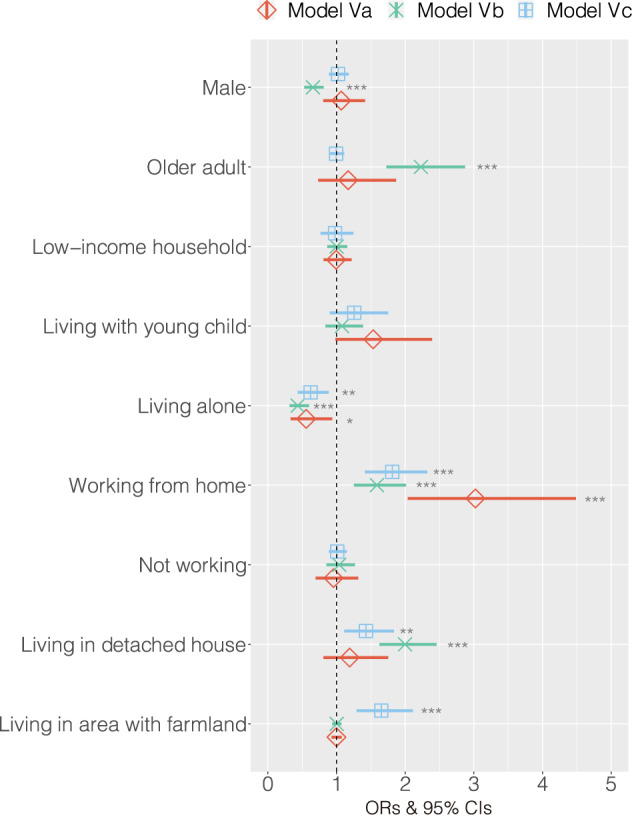
The associations between local food access and sociodemographic characteristics. Allotment farm users (**a**), home gardeners (**b**), and farm stand users (**c**). The diamond (◊), cross (x), and square (□) symbols indicate the ORs of allotment farms, home gardens, and farm stands, respectively. The vertical dashed line is the reference line and is set to one. The error bars represent the 95% CIs. Predictors are interpreted to be significant if their 95% CIs do not cross one line. The significant results are shown as follows: *p < 0.05, **p < 0.01, and ***p < 0.001.

Those who used allotment farms, home gardens, and farm stands had both common and different characteristics (Fig. 5 and Supplementary Table 8). First, the common attributes that were positively and significantly associated with all types of access were not living alone and working from home. Second, the attributes specific to each were being women, being older people, living in a detached house for home gardens and living in a detached house, and living in an area with farmlands for farm stands. No additional attributes for allotment farms were observed.

Characteristically, working from home, which became a common work style after the COVID-19 pandemic, showed high ORs for all types: allotment farms (OR: 3.02, CI: 2.04, 4.49, *p* < 0.001), home gardens (OR: 1.59, CI: 1.25, 2.01, *p* < 0.001), and farm stands (OR: 1.81, CI: 1.41, 2.32, *p* < 0.001). In contrast, being in a low-income household was not significantly associated with any type of local food access, indicating that a low-income level did not limit access to local fresh foods.

## Discussion

During the COVID-19 pandemic, behavioral restrictions were imposed, after which various health problems were reported in many countries^45,46^. The pandemic has also increased food insecurity worldwide; consequently, panic buying has been observed in many countries, including Japan^47^. However, even in such situations, we found that diversity in local food access, ranging from self-cultivation to direct-to-consumer sales, was significantly associated with health and food security variables. Specifically, our results revealed the following five key discussion points.

### Urban agriculture in walkable neighborhoods bore fruit for health and food system resilience. However, the magnitude of its contribution differed depending on the type of urban agriculture

The results of this study showed that those who grew food by themselves at allotment farms and home gardens had significantly better subjective well-being and physical activity levels than those who did not. This result is in line with previous studies conducted during times free from the impact of infectious disease pandemics^38–40^. The use of direct sales was not related to subjective well-being but was significantly associated with physical activity. The reason might be that farm stand users tend to live in areas with farmland and travel to purchase fruits and vegetables at farm stands on foot or by bicycle. This result is consistent with that of a previous study demonstrating that the food environment in neighborhoods is an important component in promoting physical activity^17^.

Our results also showed that those who grew food by themselves at allotment farms and those who purchased local foods at farm stands were significantly less anxious about the availability of fresh food both during the state of emergency and in the future than their counterparts. In contrast, home garden users showed significant differences only for the state of emergency. This result might be due to the differences in the size and yield of cultivation at allotment farms and home gardens. One lot in allotment farms in Tokyo can produce as much as or more than the average annual vegetable consumption per household in Japan^48^. However, home gardens are generally smaller and produce limited fresh foods for consumption, which may have influenced food security concerns.

As in other countries, Japan imports much food from overseas and is deeply integrated into the large-scale global food system. However, as shown in this study, urban agriculture in Japanese suburbs forms small-scale, decentralized, and community-based local food systems. This multilayered food system can complement the disruptions and shortages of the global system when various problems occur for climatic, sociopolitical, or other reasons, such as pandemics. In fact, our empirical evidence suggests that urban agriculture in walkable neighborhoods, particularly allotment farms and direct-to-consumer sales at farm stands, contributed to the mitigation of food security concerns in neighborhood communities. This means that urban agriculture could enhance the resilience of the urban food system at a time when the global food system has been disrupted due to a pandemic. This validates recent discussions about the potential of urban agriculture to facilitate food system resilience^10^. Furthermore, our findings imply that the types of urban agriculture employed matter in determining the degree of contribution to food system resilience.

To summarize the overall results, urban agriculture in walkable neighborhoods bore fruit for health and food system resilience during the COVID-19 pandemic. However, different types of urban agriculture exhibited varying associations with health and resilience. Allotment farms were positively related to all of the following: subjective well-being, physical activity, and food security concerns, both during the state of emergency and in the future. Home gardens were positively related to subjective well-being, physical activity, and food security concerns only during the state of emergency. Farm stands were positively related to physical activity and food security concerns both during the state of emergency and in the future.

These differences may be due to the characteristics of the respective spaces. It is suggested that this diversity of urban agriculture has led to different types of people benefiting from various kinds of urban agriculture. Allotment farms were found to be associated with high subjective well-being, physical activity, and food security, but they may not be feasible for those who do not have enough physical strength because users are responsible for cultivating their lots, which measure 10–30 square meters^40^. In contrast, home gardens can be created even by those who are not confident in their physical strength. In fact, our study showed that women and older people engaged in home gardening more than men and younger people. In addition, direct-to-consumer sales at farm stands are the easiest way to obtain local fresh foods for those who do not have the time and space for allotment farms and home gardens. The need for urban agriculture has been argued in many countries^2,3^. However, little attention has been paid to its scale, accessibility, and diversity. Our study suggests that it is worthwhile to create diverse food production spaces within walkable neighborhoods while considering the diversity of people who access these spaces.

### Compared to other urban greenery and food retailers, the benefits of urban agriculture on subjective well-being and food security could be greater

Compared to the use of other urban green spaces, including urban parks, our results indicated that self-cultivation at allotment farms and home gardens was more strongly associated with subjective well-being. Previous studies have offered limited perspectives on the differences among various types of urban green spaces^33^. Our study further suggests that urban parks, allotment farms, and home gardens are differently associated with human health. However, as the reason was not determined, further research is needed.

Furthermore, compared to other food retailers, such as supermarkets, convenience stores, and co-op deliveries, allotment farms and farm stands were more strongly associated with less anxiety about fresh food availability in the future. The availability of local fresh foods within walkable neighborhoods might have mitigated food security concerns because residents could grow food by themselves or directly observe farmers’ production processes, which may have made the difference from purchasing at places where the food systems were not visible.

### Flexibility in work style might promote urban agriculture in walkable neighborhoods

There was an association between work style—working from home—and access to local food. According to the Ministry of Health, Labor and Welfare (https://www.mhlw.go.jp/english), 52% of Tokyo office workers worked from home during the first emergency declaration. Long commute times and high train congestion rates have been a problem in Tokyo suburbs, but remote workers have gained more time at and around their homes by reducing their commute times, increasing their opportunities to access local food in their walkable neighborhoods. Those who worked from home sought outdoor activities for refreshment and exercise and used a variety of urban green spaces during the pandemic^49^. Allotment farms and home gardens might be used as such urban green spaces. This result is consistent with previous studies assessing the characteristics of Canadian gardeners during the COVID-19 pandemic^28,30^.

Until now, urban planners and policymakers have rarely taken work style into account. However, the flexibility of work styles and work hours may bring new insights; for example, those who work from home may become important players in urban agriculture. It has been pointed out that cities have a large hidden potential for urban agriculture by cultivating underused lands^50^. Our study suggests that such underused lands could be converted into productive urban landscapes for remote workers to engage in farming or gardening in between jobs as a hobby or as a side business.

### Food equity might be improved by urban agriculture in walkable neighborhoods

Local fresh food is generally considered more expensive than junk food in high-income countries, creating social issues of food inequity. Therefore, past discussions on urban agriculture and food security have focused primarily on low-income households in socioeconomically disadvantaged areas^24–26^.

In contrast, our study covered people from all income groups and found no statistically significant relationship between access to local food and income. This finding might be due to two urban cultural backgrounds regarding local food in Tokyo, that is, accessibility and affordability. First, residential segregation by income levels is not noteworthy in Tokyo and people from various income brackets live mixed in the same neighborhoods^51^. Therefore, most urban residents living in the suburbs have geographically equitable opportunities to access local foods. Second, local foods sold at farm stands are affordable. Prices are almost the same or cheaper than buying food at food retailers. While prices increase because of middleman margins related to shipping in the wholesale market, such increases are unnecessary when selling directly to consumers at farm stands. In addition, the allotment farm lots are not expensive to rent, particularly those operated by local municipalities (Supplementary Note 1).

These two backgrounds make local fresh food physically and economically accessible to consumers of all income levels, resulting in food equity. This is particularly important because the concept of food system resilience includes the equitability perspective^27^.

### The integration of urban agriculture into walkable neighborhoods is a fruitful way

While the current discussion on walkable neighborhoods does not emphasize urban agriculture, our evidence indicated its effectiveness. The concept of walkable neighborhoods (e.g., the 15-min city model) stresses the decarbonization benefit of limiting vehicle travel, as well as the health benefits of promoting walking and cycling^13–16^. In addition, our research indicated that urban agriculture in walkable neighborhoods benefited health and well-being by increasing recreational outdoor opportunities to neighborhood communities, including remote workers. It also contributed to food system resilience by providing local foods to all people, including low-income households, when the global food system was disrupted due to the pandemic. Furthermore, recent studies on urban agriculture reported the decarbonization benefit of reducing carbon footprints in food production and distribution^7,8^. Small-scale and community-based urban agriculture in walkable neighborhoods might especially bring this benefit because neighborhood communities travel to farms on foot or by bicycle, which means almost no emission by distribution. While urban green spaces have various health benefits^32–35^, urban agriculture also contributes to food system resilience as well as carbon emission reduction, which makes it unique.

Urban agriculture was once considered a failure of urban planning in Japan because it symbolized uncontrolled sprawl. This is analogous to the Western view, as urban agriculture was once considered the ultimate oxymoron^1^. However, our empirical evidence suggests that the urban‒rural mixture at neighborhood scales is a reasonable urban form that contributes to the resilience of the urban food system and to the health and well-being of neighborhood communities. It is no longer a failure of urban planning but a legacy of urban sprawl in the current urban context.

Our study showed that integrating urban agriculture into walkable neighborhoods is a fruitful way of creating healthier cities and developing more resilient urban food systems during times of uncertainty. In cities where there is no farmland in intraurban areas, it would be considered effective to utilize underused spaces such as vacant lots and rooftops as productive urban landscapes. In growing cities where urban areas are still expanding, it would be advantageous to conserve agricultural landscapes within their urban fabrics. Our study could provide referential insights and robust evidence for urban policy to integrate urban agriculture into walkable neighborhoods.

This study has potential limitations, including the timing of the survey and the measurement method that was utilized. We conducted the survey between June 4 and 8, 2020, just after the end of the first declaration of a state of emergency by the Japanese government. During this period, the main cultivation activities were planting and growing, and the harvest was just beginning. This seasonal constraint may have influenced the results. Because the survey was conducted during the pandemic, we used subjective methods to measure health and well-being status. However, the results might be different using objective methods^52^, thus further research is necessary. In addition, a longitudinal study is needed to determine whether the trends observed in this study were specific to the emergency period or whether they will persist after the COVID-19 pandemic.

## Methods

### Data sampling

An online questionnaire survey was conducted using the platform provided by Macromill, Inc., an online research company. An online survey was the most feasible option during the COVID-19 emergency. A total of 4126 people living in Tokyo, aged 20 years and above, responded to the questionnaire. The number of questionnaire respondents was adjusted in advance to include at least 1000 people who were experienced in using allotment farms and/or farm stands. Among all the respondents, 1030 (25%) were experienced, and 3096 (75%) were not. The number of respondents was adjusted during the recruitment process so that they would be evenly distributed across age groups (i.e., divided based on those in their 20, 30, 40, 50, 60, and 70 s and above) and places of residence (i.e., Tokyo special wards and Tama suburban cities). Among Tokyo municipalities, towns and villages were excluded from the survey, as they have a smaller population size and a lower density than wards and cities. After excluding the questionnaires of those with missing data (i.e., those who did not answer the income question, those who did not indicate their neighborhood, and those who did not properly complete the short version of the International Physical Activity Questionnaire), the analytical sample comprised 3135 participants.

The survey was conducted between June 4 and 8, 2020, shortly after the end of the first declaration of a state of emergency by the Japanese National Government, which remains the most severe measure the government has taken under the law. During this period, while people were requested to refrain from unnecessarily leaving their homes, shopping for daily goods, hospital visits, and outdoor exercise were not restricted. People could also actively engage in urban agriculture. At most allotment farms, they were able to engage in farming activities as long as they took infectious disease control measures. Public interest in home gardening also increased (Supplementary Note 1). Professional farmers continued sales at farm stands, and neighborhood communities were able to purchase fresh local food.

The survey questions were classified into three categories (Supplementary Method 1). The participants also had to answer basic questions on sociodemographic factors. The first category of questions concerned access to local food and related facilities. The respondents were asked about the frequency of access during the state of emergency to allotment farms, home gardens (including terraces and balconies), and farm stands on a five-point scale (almost every day, three or four times a week, one or two times a week, a few times a month, no access). To highlight those who grew food in their home gardens, we asked a separate question on this point. We also asked about the frequency of access to other food purchasing sites (e.g., supermarkets, convenience stores, and co-op deliveries) and other urban green spaces (e.g., small parks, large parks, and greenways) on the same five-point scale for comparison (Supplementary Fig. 2). To identify those who regularly accessed local food during the state of emergency, those who used it at least one or two times a week were selected as users of each category. Then, it was found that there were 147 allotment users, 497 home gardeners, and 326 farms stand users.

The second category concerned subjective well-being and physical activity. To evaluate subjective well-being, we employed the World Health Organization Five Well-Being Index (WHO-5), one of the most widely used questionnaires for assessing subjective psychological well-being^53^. Particularly, we used the S-WHO-5-J, which was developed for Japanese respondents; its reliability and validity have been examined in past research^54^. For physical activity, we employed the short version of the IPAQ, which was developed for cross-national monitoring of physical activity^55^. The time periods set by the original questions are “over the last 2 weeks” for the WHO-5 and ‘in the last seven days’ for the short version of the International Physical Activity Questionnaire (IPAQ). However, in our questionnaire, we set the time period to ‘during the period when the state of emergency was declared.’ Two methods of measuring health and well-being can be employed: objective and subjective^35,52^. Objective methods include the measurement of individuals’ health status by investigators using instruments; however, this was impossible to undertake during the pandemic when direct human contact was highly restricted. Therefore, we used a subjective method, in which we asked about individuals’ perceptions using the existing common indicators.

The third category was related to food security concerns. Existing indicators of food security have been developed primarily for low-income households. However, they are not appropriate for this paper since the purpose of this study was to understand the food-related concerns of urban residents, rich and poor alike, in the context of the COVID-19 pandemic. Therefore, we simply asked the respondents to rate their food security concerns, particularly their anxiety about the availability of fresh food, such as vegetables and fruits, during the state of emergency and if logistics were to be disrupted in the future on a five-point Likert scale (strongly disagree, disagree, neither agree nor disagree, agree, strongly agree). Since the survey was conducted at a time of high uncertainty and unpredictability, we asked about the state of emergency and the future separately.

The collected sociodemographic factors included the respondent’s gender, age, household income, family structure (living alone, living with young children, and living with someone but not young children), employment status (not working, working from home, and working at an office), housing type (living in a detached house and living in an apartment complex), and neighborhood of residence. Regarding employment status, working from home supported by digital transformation became widespread during the COVID-19 pandemic. Therefore, we included this work style in the questionnaire.

This study was approved by the Ethical Committee of the Graduate School of Engineering, The University of Tokyo (approval number: KE20-8). Informed consent was obtained from all respondents. However, because it was an online questionnaire survey, the consent was not written paper. The survey was completely anonymous and confidential. All respondents were informed of their right to withdraw at any time.

### Spatial analysis

To distinguish between the impact of access to local food and that of the existence of food production spaces in the regression analysis described below, we calculated the percentage of farmland in neighborhoods called “*Cho-cho-moku*” (Supplementary Fig. 3). The presence or absence of farmland in neighborhoods was then incorporated as an attribute of the survey respondents. “*Cho-cho-moku*” is a basic spatial division for residential indication in Japan and is also used for the national census. ArcGIS Pro 2.9 was used for the analysis.

### Statistical analysis

We performed logistic regression analyses using the cross-sectional dataset of 3135 adults (Supplementary Fig. 1 and Supplementary Tables 2, 3). First, binomial logistic regression analyses were conducted to determine the relationship between access to local food and subjective well-being (Model 1a) and physical activity (Model 2a). As allotment farms and home gardens are types of urban green spaces, the variables of other types of urban green spaces (small parks, large parks, and greenways) were also included in the other regression models (Model 1b and Model 2b). Additionally, we developed extra modeling structures to compare access to local food and other urban green spaces, including the factors showing the strongest relationship with subjective well-being (large parks) and physical activity (greenways), against allotment farms (Model 1c and 2c), home gardens (Model 1d and 2d), or farm stands (Model 1e and 2e). We used the S-WHO-5-J score for the outcome of subjective well-being, and those whose scores were less than half were classified in the “poor” well-being status category; the rest were considered to be under the “fine” status category, using the WHO-5 score criteria as a reference^53,54^. For physical activity, we calculated the total physical activity per week [the metabolic equivalent of task (MET)-minutes/week] using the IPAQ short version. Those who exceeded the level recommended by the Ministry of Health, Labor and Welfare (18–64 years: 1380 MET-minutes/week; ≥65 years: 600 MET-minutes/week) were then considered “active” in terms of physical status; the rest were considered “not active.”

Second, ordinal logistic regression analyses were conducted to observe the relationship between the three types of local food access and food security concerns during the state of emergency (Model 3a) and in the future (Model 4a). The variables of other types of food purchasing places (supermarkets, convenience stores, and co-op deliveries) were also included in the other regression models (Models 3b and 4b). Additionally, we developed extra modeling structures to compare access to local food and other food purchasing places, including the factors showing the strongest relationship with food security concerns during the state of emergency (supermarkets) and in the future (convenience stores), against allotment farms (Model 3c and 4c), home gardens (Model 3d and 4d), or farm stands (Model 3e and 4e).

All models were adjusted for sociodemographic control variables that could be potential confounders: gender, age, income, family structure, work status, housing type, and neighborhood environment. In particular, we added working from home as a work status variable because that was a new work style significantly affected by the COVID-19 pandemic.

Furthermore, binomial logistic regression analyses were conducted to identify the characteristics of those who accessed local food: allotment farms (Model 5a), home gardens (Model 5b), and farm stands (Model 5c). The explanatory variables of these models were the sociodemographic factors shown above.

To identify potential multicollinearity among the explanatory variables, we used Pearson’s correlation coefficients for each model and the variance inflation factor for the binomial logistic regression models^56^. We produced all subsets of models, ranked them by the Akaike information criterion, and retained all models in which the ΔAkaike information criterion was <2^57^. We then calculated the averaged parameter estimates and standard errors using model averaging to ameliorate the effect of uninformative parameters^58^. Finally, we calculated the ORs and 95% CIs and plotted them. The significance level was set at *p* < 0.05. R version 4.0.2 software was used for analysis and plotting, along with the packages MASS, MuMln, and Tidyverse.

### Reporting summary

Further information on research design is available in the Nature Research Reporting Summary linked to this article.

## Supplementary information

Supplementary Materials - Final version

Reporting Summary

## Data Availability

The study population data that were used for statistical analyses are not publicly available. Based on the ethical approval granted, only those registered as coresearchers can handle the data. To request access to the data, contact the corresponding author, A.I. The spatial data on farmlands (land use) and buildings in Tokyo that were used for geographic information system (GIS) analyses were provided by the Tokyo Metropolitan Government Basic Urban Planning Survey in 2016‒2017. These data were not open data but can be used for research purposes (https://www.toshiseibi.metro.tokyo.lg.jp/seisaku/tochi_c/index.html). Other spatial data were publicly available. The data on the neighborhood division called “*Cho-cho-moku*” are available at e-Stat (e-stat.go.jp/gis), and the data on the municipality division and UPAs are available at the National Land Information Division (nlftp.mlit.go.jp/ksj).

## Code availability

The R code for our statistical analyses is available at https://github.com/AkikoII/UA-in-Tokyo-under-COVID-19/.
