# Supplementary material for: Urban agriculture in walkable neighborhoods bore fruit for health and food system resilience during the COVID-19 pandemic
Source: NPJ Urban Sustain. 2023 Feb 1;3(1):4. doi: 10.1038/s42949-023-00083-3 (PMC9890428; doi:10.1038/s42949-023-00083-3)
Supplement: Supplementary file 1 — Supplementary Materials - Final version [file 42949_2023_83_MOESM1_ESM.pdf]

Supplementary Information

Urban agriculture in walkable neighborhoods bore fruit for health and food system resilience during the COVID-19 pandemic

Akiko Iida[1] *, Takahiro Yamazaki[1, 2], Kimihiro Hino[1], Makoto Yokohari[1]

1. Department of Urban Engineering, Graduate School of Engineering, The University of Tokyo, Tokyo 1138656, Japan
2. Department of Environmental Design, Kobe Design University, Hyogo 6512196, Japan

* Corresponding author: Akiko Iida, The University of Tokyo; email: iida@epd.t.u-tokyo.ac.jp

Contents

Supplementary Note 1 | Overview of urban agriculture in Tokyo 2
Supplementary Method 1 | Questionnaire 5
Supplementary Table 1 | Farmlands in the Urbanization Promotion Areas (UPAs) in Tokyo 7
Supplementary Table 2 | Explanation of variables 8
Supplementary Table 3 | Summary statistics of variables 9
Supplementary Table 4 | ORs and 95% CIs for the association between subjective well-being and access to local food (Model 1a) and access to other urban green spaces (Model 1b) and between physical activity and access to local food (Model 2a) and access to other urban green spaces (Model 2b) 11
Supplementary Table 5 | ORs and 95% CIs for the comparison of access to large parks vs access to local food for subjective well-being (Model 1c-1e) and access to greenways vs access to local food for physical activity (Model 2c-2e) 12
Supplementary Table 6 | ORs and 95% CIs for the association between food security concerns during the state of emergency and access to local food (Model 3a) and access to other food purchasing sites (Model 3b) and food security concerns in the future and access to local food (Model 4a) and access to other food purchasing sites (Model 4b) 13
Supplementary Table 7 | ORs and 95% CIs for the comparison of access to supermarkets vs access to local food for food security concerns during the state of emergency (Model 3c-3e) and access to convenience stores vs access to local food for food security concerns in the future (Model 4c-4e) 14
Supplementary Table 8 | ORs and 95% CIs for the association between sociodemographic status and access to local food: Allotment (Model 5b), home garden (Model 5b), and farm stand (Model 5c) 15
Supplementary Figure 1 | Structure of regression models 16
Supplementary Figure 2 | Other urban green spaces and other food purchasing sites 17
Supplementary Figure 3 | Spatial analysis of farmland in Tokyo 18

Supplementary Note 1 | Overview of urban agriculture in Tokyo

Tokyo, the capital of Japan, is one of the largest cities in the world, with a population of 13.99 million. In the Urbanization Promotion Areas (UPAs) of Tokyo, which is designated by City Planning Law as the areas that are already urbanized or should be urbanized within approximately the next 10 years, farmlands take up an area of 43.1 km^2, constituting 3.9% of the total area (Fig. 1, Supplementary Table 1). In a narrow sense, farmland within the UPAs is considered as urban farmland in Japan.

These urban-rural mixed landscapes are the result of the expansion of urban areas during the period of rapid economic growth in Japan. Such urban farmlands once symbolized the uncontrolled sprawl of urban areas; subsequently, they came to be regarded as a failure of urban planning. This is analogous to the Western view, with urban agriculture once considered the ultimate oxymoron[1]. However, the 2015 Urban Farming Promotion Basic Act has led to a major policy shift, and the importance of conserving urban farmlands is now being emphasized.

The following three types are the most common forms of urban agricultural activities, especially horticultural activities, widely found in Tokyo. Allotment farming and commercial farming are practiced in the urban farmlands referred to above, which are mostly owned by farmers. Home gardening is mostly practiced on the private properties of urban residents.

1) Allotment farms

There are mainly two types of allotment farms in Japan: citizen's farms and experience farms. Citizen's farms are called '*shimin noen*' in Japanese. In most cases, municipal governments or other entities such as Japan Agricultural Cooperatives, nonprofit organizations, or private companies rent the land from farmers, open citizen's farms, and rent out lots to urban residents. In some cases, farmers open and manage citizen's farms by themselves. Experience farms are called '*taiken noen*' in Japanese. Here, farmers themselves teach urban residents the process of farming. Like citizen's farms, experience farms allot parcels of land to individuals.

According to the Tokyo Metropolitan Agricultural Promotion Office (https://www.agri.metro.tokyo.lg.jp/production/system/business/experience/individual.html?entry_id=352), there were 29,518 lots at 536 allotment farms in Tokyo in 2019, including both citizen's farms and experience farms. The majority of these, 29,034 lots at 528 allotment farms, were in the target area (i.e., Tokyo special wards and Tama suburban cities). The annual price for rent is approximately ¥5,000 to ¥50,000, the equivalent of approximately US$36 to US$357 per the US$1=¥140 conversion[2]. Most citizen's farms are located on private

[1] Morgan, K. Nourishing the city: The rise of the urban food question in the Global North. *Urban Studies* 52(8), 1379–1394 (2015).
[2] Harada, K. et al. How does urban farming benefit participants' health? A case study of allotments and experience farms in Tokyo. *International Journal of Environmental Research and Public Health* 18(2), 542 (2021).

properties owned by urban farmers but managed by local municipalities. The prices of citizen's farms are less expensive than experience farms operated by urban farmers.

Additionally, a nationwide survey by the Ministry of Agriculture, Forestry, and Fisheries identified the time and distance from homes to allotments (https://www.maff.go.jp/j/finding/mind/index.html). According to the survey, it takes less than 15 minutes for 81% of people to get to allotment farms, and 67% of people access allotment farms on foot or by bicycle in the Kanto region. Given that Tokyo has a prominent land use density, in the Kanto region, those percentages will be even higher. A study that identified the attributes of five allotment farm users in Tokyo showed that 18–34% of respondents went to the farms on foot and 50–78% went by bicycle[3].

Recently, other types of allotment farms managed by private companies as their profitable businesses have been created. For example, railroad companies have opened rental allotments on top of their station buildings in city centers such as Shinjuku and Ebisu. Venture companies have also started rental allotment services on vacant spaces of residential land. These new businesses are expected to expand in the future.

2) Home gardens

Home gardening is a popular activity in Japan. The interest in it is growing in the wake of the COVID-19 pandemic. For instance, according to the September 2020 survey by the Tokyo Metropolitan Government (https://www.metro.tokyo.lg.jp/tosei/hodohappyo/press/2020/11/19/documents/19.pdf), 34.3% of respondents practice gardening (growing mainly inedible plants) or home gardening (growing mainly edible plants) and 23.8% of respondents became interested in growing edible plants in home gardens or allotment farms during the COVID-19 pandemic.

3) Commercial farming - Direct-to-consumer sales at farm stands (*choku bai jyo*)

According to the Census of Agriculture and Forestry in Japan (https://www.maff.go.jp/e/data/stat/), there are 5,117 agricultural entities in Tokyo, of which 4,438 are in the target area (i.e., Tokyo special wards and Tama suburban cities). Among the 4,438 agricultural entities, 84% are facilitated by small-scale farmers with less than 10,000 m^2 of farmlands, most of which are operated as family businesses. Although land prices are expensive in Tokyo, tax incentives on urban farmlands (e.g., the Productive Green Land Act) have allowed farm families to continue agricultural activities even in high-density urban neighborhoods.

Agricultural enterprises ship their products to wholesale markets through supply chains, but another common way is direct-to-consumer sales. The place where farmers sell their products directly to consumers is referred to in

[3] Yagi, H. Site location and demand for the farm experience business in urban areas: An empirical analysis in Tokyo Metropolis Prefecture. *Journal of Rural Planning Association* 32, 323–328 (2013).

Japanese as ‘*choku bai jyo*,’ meaning direct-purchase place. The direct sales to consumers at ‘*choku bai jyo*’ are counted in two separate categories. One is small farm stands set up by farmers on their own land. The other type is large farm stands run by farmers’ groups in collaboration.

The detailed number of agricultural entities by sales methods is listed in the 2020 Census of Agriculture and Forestry (https://www.maff.go.jp/j/tokei/census/afc/2020/). According to the census, 71.4% of agricultural entity sellers located in the wards and cities in Tokyo sold their produce directly to consumers, with 60% at farm stands on their own farms and 25.7% at farm stands in other places, including farm stands jointly run by farmers’ groups. Only 30% of agricultural entity sellers shipped to market through Japan Agricultural Cooperatives. However, the overall trend across the country is the opposite. While 72% of agricultural entity sellers shipped to market through Japan Agricultural Cooperatives, only 21.1% sold directly to consumers, with 20% on their own farms and 42.2% in other locations. Compared to the overall trend in Japan, the wards and cities of Tokyo have a high ratio of agricultural entities that sell directly to consumers, especially on their own lands.

In other cities around the world, urban commercial agriculture is predominantly marketed through supply chains. In Tokyo, however, direct-to-consumer sales have managerial advantages and are beneficial owing to the spatial characteristics of the urban area, where farmlands are located in densely inhabited urban neighborhoods; thus, many consumers live around the farmlands.

Supplementary Method 1 | Questionnaire

1) Access to local food and related facilities

Question 1: How often did you use the following urban green spaces during the period when the state of emergency was declared?

A) Allotment farm
B) Home garden (including terrace and balcony)
C) Small park (with some playground equipment and benches)
D) Large park (with large open spaces of some size and sports facilities)
E) Greenway (including riverside path)
F) Woodland and thicket
G) Temple and shrine

Answer: Five-point scale (1) Almost every day, (2) Three or four times a week, (3) One or two times a week, (4) A few times a month, (5) No access

Question 2: How often did you use the following food purchasing sites to buy vegetables and fruits during the period when the state of emergency was declared?

A) Direct-to-consumer sales at farm stand (*choku bai jyo*)
B) Supermarket
C) Convenience store
D) Coop-delivery
E) Greengrocer
F) Department store
G) Online store

Answer: Five-point scale (1) Almost every day, (2) Three or four times a week, (3) One or two times a week, (4) A few times a month, (5) No access

Question 3: Did your activity regarding the following matters change during the period when the state of emergency was declared?

A) Growing food at allotment farm
B) Growing food in home garden
C) Purchasing food at direct-to-consumer sales at farm stand (*choku bai jyo*)

Answer: Six options (1) Started newly, (2) Frequency was increased, (3) Frequency was decreased, (4) Frequency was not changed, (5) Don’t use but interested, (6) Don’t use and not interested

2) Subjective well-being and physical activity

Question 4: Simplified Japanese version of the WHO-Five Well-Being Index (S-WHO-5-J)[4]

Question 5: Short version of the International Physical Activity Questionnaire (IPAQ)[5]

3) Food security concerns during the state of emergency and in the future

Question 6: How did you feel about purchasing or obtaining vegetables and fruits during the period when the state of emergency was declared?

A) I have no concerns about purchasing or obtaining vegetables and fruits.

B) I would have no concerns about purchasing or obtaining vegetables and fruits if logistics were to be disrupted in the future.

Answer: Five-point scale (1) Strongly disagree, (2) Disagree, (3) Neither agree nor disagree, (4) Agree, (5) Strongly agree

[4] Inagaki, H. et al. Reliability and validity of the simplified Japanese version of the WHO-Five Well-Being Index (S-WHO-5-J). *Japanese Journal of Public Health* 60(5), 294–301 (2013).

[5] Craig, C. L. et al. International Physical Activity Questionnaire: 12-country reliability and validity. *Medicine and Science in Sports and Exercise* 35(8), 1381–1395 (2003).

Supplementary Table 1 | Farmlands in the Urbanization Promotion Areas (UPAs) in Tokyo

	**Area (km^2)**	**Area (%) in the UPAs**
Farmlands in the UPAs	43.1	3.9%
0.5 km buffer zone of farmlands in the UPAs	805.1	73.4%
1.0 km buffer zone of farmlands in the UPAs	926.6	84.4%
Urbanization Promotion Areas (UPAs)	1097.3	100%

Urbanization Promotion Areas (UPAs): Areas that are already urbanized or should be urbanized within approximately the next 10 years, designated by local municipalities based on the City Planning Law. Farmland areas were calculated by ArcGIS Pro 2.9 using the land use data of the Tokyo Metropolitan Government Basic Urban Planning Survey in 2016–2017.

Supplementary Table 2 | Explanation of variables

Based on the questions described in Supplementary Method 1, each variable was set as follows:

Variables	Explanation
**Response variables**	
Subjective well-being[*1] (Models 1a-1e)	1: Persons whose S-WHO-5-J scores were half or more in Question 4. Their well-being status was 'fine.'<br>0: Persons whose S-WHO-5-J scores were less than half in Question 4. Their well-being status was 'poor.'
Physical activity[*1] (Models 2a-2e)	1: Persons whose total physical activity per week in Question 5 exceeded the level recommended by the national government. Their physical status was 'active.'<br>0: Persons whose total physical activity per week in Question 5 was below the level recommended by the national government. Their physical status was 'not active.'
Food security concerns during the state of emergency (Models 3a-3e)	5-point scale in Question 6-A)
Food security concerns in the future (Models 4a-4e)	5-point scale in Question 6-B)
**Explanatory variables**	
Access to local food	
Allotment farm	1: Persons who answered (1), (2), or (3) in Question 1-A)<br>0: Persons who answered other portions
Home garden[*2]	1: Persons who answered (1), (2), or (3) in Question 1-B) and besides who answered (1), (2), (3), or (4) in Question 3-B)<br>0: Persons who answered other portions
Farm stand	1: Persons who answered (1), (2), or (3) in Question 2-A)<br>0: Persons who answered other portions
Access to other urban green spaces[*3]	
Small park	1: Persons who answered (1), (2), or (3) in Question 1-C)<br>0: Persons who answered other portions
Large park	1: Persons who answered (1), (2), or (3) in Question 1-D)<br>0: Persons who answered other portions
Greenway	1: Persons who answered (1), (2), or (3) in Question 1-E)<br>0: Persons who answered other portions
Access to other food purchasing sites[*4]	
Supermarket	1: Persons who answered (1), (2), or (3) in Question 2-B)<br>0: Persons who answered other portions
Convenience store	1: Persons who answered (1), (2), or (3) in Question 2-C)<br>0: Persons who answered other portions
Coop-delivery	1: Persons who answered (1), (2), or (3) in Question 2-D)<br>0: Persons who answered other portions

*1: The calculation methods are explained in the manuscript methods.

*2: The two questions were combined to extract the persons who grow food in their home gardens.

*3: Among five types of other urban green spaces from Question 1-C) to Question 1-G), three categories that had the most users were selected.

*4: Among six types of other food purchasing sites from Question 2-B) to Question 1-G), three categories that had the most users were selected.

Supplementary Table 3 | Summary statistics of variables

Presented here are summary statistics of response variables, explanatory variables, and control variables of Models 1, 2, 3, and 4 (n=3,135)

			n	%
**Response variables**				
	Subjective well-being (Models 1a-1e)	1: Fine	1,538	49.1%
		0: Poor	1,597	50.9%
	Physical activity (Models 2a-2e)	1: Active	1,583	50.5%
		0: Not active	1,552	49.5%
	Food security concerns during the state of emergency (Models 3a-3e)	1: Strongly disagree	63	11.5%
		2: Disagree	284	50.3%
		3: Neither	853	27.2%
		4: Agree	1,576	9.1%
		5: Strongly agree	359	2.0%
	Food security concerns in the future (Models 4a-4e)	1: Strongly disagree	207	6.6%
		2: Disagree	725	23.1%
		3: Neither	1,298	41.4%
		4: Agree	741	23.6%
		5: Strongly agree	164	5.2%
**Explanatory variables**				
**Access to local food**				
	Allotment farm	1: User	147	4.7%
		0: Not a user	2,988	95.3%
	Home garden	1: User	497	15.9%
		0: Not a user	2,638	84.1%
	Farm stand	1: User	326	10.4%
		0: Not a user	2,809	89.6%
Access to other urban green spaces				
	Small park	1: User	604	19.3%
		0: Not a user	2,531	80.7%
	Large park	1: User	429	13.7%
		0: Not a user	2,706	86.3%
	Greenway	1: User	699	22.3%
		0: Not a user	2,436	77.7%
Access to other food purchasing sites				
	Supermarket	1: User	2,672	85.2%
		0: Not a user	463	14.8%

Convenience store	1: User	1,305	41.6%
	0: Not a user	1,830	58.4%
Co-op delivery	1: User	556	17.7%
	0: Not a user	2,579	82.3%
**Explanatory variables (sociodemographic characteristics) as control variables**			
Gender	1: Male	1,829	58.3%
	0: Female	1,306	41.7%
Older adult	1: ≥ 65 years old	710	22.6%
	0: < 65 years old	2,425	77.4%
Low-income household*1	1: < ¥2,000,000 /year	216	6.9%
	0: ≥ ¥2,000,000 /year	2,919	93.1%
Living alone	1: Yes	732	23.3%
	0: No	2,403	76.7%
Living with young children*2	1: Yes	517	16.5%
	0: No	2,618	83.5%
Working from home*3	1: Yes	1,350	43.1%
	0: No	1,785	56.9%
Not working	1: Yes	921	29.4%
	0: No	2,214	70.6%
Living in detached house	1: Yes	1,214	38.7%
	0: No	1,921	61.3%
Living in area with farmland*4	1: Yes	1,507	48.1%
	0: No	1,628	51.9%

*1: Low-income household: The survey asked about household income in 2-million-yen increments. In this paper, a low-income household means a household whose income is lower than ¥2,000,000, approximately US$14,286 per the conversion US$1 = ¥140. This threshold was set based on the Japanese relative poverty household income (¥1,270,000), determined by the Ministry of Health, Labour and Welfare (https://www.mhlw.go.jp/toukei/saikin/hw/k-tyosa/k-tyosa19/).
*2: Living with young children: Families whose ages are 20s to 40s and living with children.
*3: Working from home: People who work from home at least one day a week.
*4: The dummy variable ‘Living in an area with farmlands’ was defined as those who live in neighborhoods (*cho-cho-moku*) with more than 1% area of farmlands.

Supplementary Table 4 | ORs and 95% CIs for the association between subjective well-being and access to local food (Model 1a) and access to other urban green spaces (Model 1b) and between physical activity and access to local food (Model 2a) and access to other urban green spaces (Model 2b)

	Subjective well-being						Physical activity					
	Model 1a			Model 1b			Model 2a			Model 2b		
Variables	ORs	(95% CIs)	*P*	ORs	(95% CIs)	*P*	ORs	(95% CIs)	*P*	ORs	(95% CIs)	*P*
(Intercept)	0.85	(0.73, 1.00)	0.04*	0.85	(0.72, 1.00)	0.05	0.93	(0.77, 1.13)	0.48	0.82	(0.72, 0.93)	0.002**
Allotment farm	2.03	(1.38, 2.98)	<0.001 ***				2.25	(1.48, 3.40)	<0.001 ***			
Home garden	1.51	(1.23, 1.86)	<0.001 ***				1.76	(1.43, 2.18)	<0.001 ***			
Farm stand	1.12	(0.86, 1.47)	0.39				2.06	(1.58, 2.68)	<0.001 ***			
Small park				1.22	(0.96, 1.56)	0.10				1.68	(1.34, 2.11)	<0.001 ***
Large park				1.38	(1.08, 1.76)	0.009**				1.98	(1.52, 2.59)	<0.001 ***
Greenway				1.13	(0.91, 1.41)	0.28				3.36	(2.73, 4.14)	<0.001 ***
Male	0.85	(0.73, 0.99)	0.035*	0.83	(0.71, 0.96)	0.01*	1.10	(0.92, 1.31)	0.30	1.01	(0.94, 1.09)	0.83
Older adult	1.30	(1.08, 1.58)	0.007**	1.33	(1.10, 1.61)	0.004**	0.61	(0.50, 0.75)	<0.001 ***	0.60	(0.49, 0.74)	<0.001 ***
Low-income household	0.67	(0.49, 0.91)	0.009**	0.68	(0.51, 0.93)	0.01*	0.74	(0.55, 0.99)	0.04*	0.92	(0.69, 1.21)	0.55
Living alone	0.80	(0.67, 0.96)	0.01*	0.78	(0.65, 0.94)	0.009**	1.00	(0.97, 1.03)	0.98	0.99	(0.92, 1.07)	0.87
Living with young child	1.00	(0.93, 1.06)	0.90	0.97	(0.84, 1.12)	0.69	1.00	(0.97, 1.04)	0.97	0.92	(0.74, 1.15)	0.48
Working from home	1.35	(1.15, 1.59)	0.001 ***	1.36	(1.16, 1.60)	<0.001 ***	1.09	(0.90, 1.32)	0.36	1.00	(0.97, 1.04)	0.97
Not working	0.98	(0.88, 1.10)	0.79	0.99	(0.89, 1.10)	0.78	0.92	(0.74, 1.14)	0.43	0.80	(0.66, 0.96)	0.02*
Living in detached house	1.01	(0.93, 1.10)	0.80	1.06	(0.91, 1.24)	0.46	0.98	(0.87, 1.09)	0.67	1.00	(0.96, 1.05)	0.93
Living in area with farmland	1.00	(0.95, 1.04)	0.91	1.00	(0.97, 1.03)	0.95	0.94	(0.81, 1.10)	0.46	0.99	(0.91, 1.07)	0.79

***p<0.001; **p<0.01; *p<0.05; ORs: odds ratios; CIs: confidence intervals

Supplementary Table 5 | ORs and 95% CIs for the comparison of access to large parks vs access to local food for subjective well-being (Model 1c-1e) and access to greenways vs access to local food for physical activity (Model 2c-2e)

	Subjective well-being						Physical activity					
	Model 1c		Model 1d		Model 1e		Model 2c		Model 2d		Model 2e	
Variables	ORs (95% CIs)	*P*	ORs (95% CIs)	*P*	ORs (95% CIs)	*P*	ORs (95% CIs)	*P*	ORs (95% CIs)	*P*	ORs (95% CIs)	*P*
(Intercept)	0.88 (0.75, 1.03)	0.12	0.83 (0.71, 0.98)	0.03*	0.87 (0.74, 1.02)	0.10	0.89 (0.78, 1.01)	0.07	0.85 (0.73, 0.99)	0.03*	0.89 (0.78, 1.02)	0.10
Allotment farm	2.06 (1.41, 3.00)	<0.001***					2.23 (1.46, 3.39)	<0.001***				
Home garden			1.59 (1.30, 1.95)	<0.001***					1.84 (1.49, 2.28)	<0.001***		
Farm stand					1.34 (1.05, 1.70)	0.02*					1.94 (1.49, 2.54)	<0.001***
Large park	1.44 (1.16, 1.79)	0.001**	1.54 (1.24, 1.90)	<0.001***	1.52 (1.23, 1.89)	<0.001***						
Greenway							4.26 (3.50, 5.20)	<0.001***	4.36 (3.58, 5.31)	<0.001***	4.21 (3.45, 5.13)	<0.001***
Male	0.83 (0.71, 0.96)	0.01	0.85 (0.73, 0.99)	0.04*	0.83 (0.71, 0.97)	0.02*	1.02 (0.92, 1.12)	0.73	1.04 (0.91, 1.19)	0.59	1.01 (0.93, 1.10)	0.78
Older adult	1.34 (1.11, 1.63)	0.003**	1.28 (1.06, 1.55)	0.01*	1.36 (1.12, 1.64)	0.002**	0.62 (0.51, 0.76)	<0.001***	0.58 (0.47, 0.72)	<0.001***	0.63 (0.51, 0.77)	<0.001***
Low-income household	0.68 (0.50, 0.92)	0.01*	0.68 (0.50, 0.92)	0.01*	0.68 (0.50, 0.92)	0.01*	0.84 (0.60, 1.17)	0.30	0.86 (0.63, 1.19)	0.37	0.86 (0.62, 1.19)	0.36
Living alone	0.78 (0.65, 0.94)	0.008**	0.80 (0.67, 0.96)	0.01*	0.78 (0.65, 0.93)	0.007**	1.00 (0.95, 1.05)	0.94	1.00 (0.96, 1.04)	0.99	0.99 (0.92, 1.07)	0.86
Living with young child	0.98 (0.88, 1.10)	0.79	0.99 (0.90, 1.09)	0.82	0.99 (0.91, 1.08)	0.84	1.00 (0.95, 1.05)	0.99	1.00 (0.96, 1.04)	0.98	1.00 (0.95, 1.05)	0.99
Working from home	1.35 (1.15, 1.59)	<0.001***	1.35 (1.15, 1.59)	<0.001***	1.36 (1.16, 1.61)	<0.001***	1.00 (0.95, 1.06)	0.91	1.00 (0.96, 1.04)	0.94	1.00 (0.95, 1.06)	0.91
Not working	0.98 (0.87, 1.11)	0.77	0.98 (0.88, 1.10)	0.77	0.98 (0.85, 1.12)	0.72	0.80 (0.67, 0.97)		0.79 (0.65, 0.95)	0.01*	0.79 (0.66, 0.96)	0.02*
Living in detached house	1.04 (0.91, 1.20)	0.56	1.01 (0.93, 1.10)	0.78	1.03 (0.91, 1.17)	0.62	1.00 (0.96, 1.04)	0.97	0.99 (0.91, 1.07)	0.79	1.00 (0.96, 1.04)	0.99
Living in area with farmland	1.00 (0.96, 1.05)	1.00	1.00 (0.95, 1.05)	0.97	1.00 (0.95, 1.05)	0.96	0.97 (0.86, 1.09)	0.62	0.94 (0.81, 1.10)	0.45	0.90 (0.76, 1.07)	0.23

$^{***}p<0.001$; $^{**}p<0.01$; $^{*}p<0.05$; ORs: odds ratios; CIs: confidence intervals

Supplementary Table 6 | ORs and 95% CIs for the association between food security concerns during the state of emergency and access to local food (Model 3a) and access to other food purchasing sites (Model 3b) and food security concerns in the future and access to local food (Model 4a) and access to other food purchasing sites (Model 4b)

	Food security concerns during the state of emergency						Food security concerns in the future					
	Model 3a			Model 3b			Model 4a			Model 4b		
Variables	ORs	(95% CIs)	*P*	ORs	(95% CIs)	*P*	ORs	(95% CIs)	*P*	ORs	(95% CIs)	*P*
Allotment farm	1.61	(1.15, 2.26)	0.006**				2.92	(2.08, 4.10)	<0.001 ***			
Home garden	1.22	(1.01, 1.48)	0.04*				1.12	(0.91, 1.38)				
Farm stand	1.48	(1.17, 1.88)	0.001**				1.92	(1.52, 2.43)	<0.001 ***			
Supermarket				1.50	(1.25, 1.81)	<0.001 ***				1.00	(0.96, 1.05)	0.97
Convenience store				1.00	(0.97, 1.04)	0.97				1.44	(1.26, 1.64)	<0.001 ***
Co-op delivery				1.01	(0.95, 1.07)	0.86				1.18	(0.98, 1.42)	0.08
Male	0.86	(0.75, 0.99)	0.04*	0.89	(0.76, 1.05)	0.18	1.07	(0.92, 1.23)	0.40	1.01	(0.95, 1.07)	0.82
Older adult	1.88	(1.58, 2.24)	<0.001 ***	1.93	(1.61, 2.31)	<0.001 ***	1.43	(1.21, 1.70)	<0.001 ***	1.51	(1.28, 1.78)	<0.001 ***
Low-income household	0.73	(0.56, 0.95)	0.02*	0.80	(0.59, 1.08)	0.14	0.63	(0.48, 0.81)	<0.001 ***	0.64	(0.49, 0.83)	0.001 ***
Living alone	0.97	(0.85, 1.11)	0.63	0.86	(0.72, 1.03)	0.09	0.96	(0.82, 1.11)	0.55	0.95	(0.80, 1.11)	0.50
Living with young child	1.00	(0.95, 1.05)	0.99	1.00	(0.96, 1.05)	0.99	1.00	(0.95, 1.06)	0.88	1.01	(0.93, 1.10)	0.81
Working from home	1.03	(0.91, 1.15)	0.66	1.12	(0.95, 1.33)	0.18	1.17	(1.01, 1.35)	0.03*	1.23	(1.07, 1.42)	0.003**
Not working	1.01	(0.92, 1.12)	0.78	1.02	(0.91, 1.15)	0.74	0.99	(0.92, 1.07)	0.84	1.00	(0.95, 1.05)	0.95
Living in detached house	0.99	(0.93, 1.06)	0.82	1.00	(0.96, 1.04)	0.95	1.03	(0.92, 1.17)	0.59	1.15	(0.98, 1.33)	0.08
Living in area with farmland	1.00	(0.97, 1.04)	0.96	1.00	(0.97, 1.04)	0.91	1.00	(0.96, 1.04)	0.89	1.01	(0.94, 1.09)	0.77
1|2	0.02	(0.02, 0.03)	<0.001 ***	0.03	(0.02, 0.04)	<0.001 ***	0.09	(0.07, 0.11)	<0.001 ***	0.10	(0.08, 0.12)	<0.001 ***
2|3	0.14	(0.12, 0.16)	<0.001 ***	0.19	(0.15, 0.23)	<0.001 ***	0.54	(0.47, 0.63)	<0.001 ***	0.61	(0.52, 0.71)	<0.001 ***
3|4	0.70	(0.61, 0.81)	<0.001 ***	0.95	(0.76, 1.19)	0.68	3.35	(2.87, 3.91)	<0.001 ***	3.68	(3.12, 4.34)	<0.001 ***
4|5	9.35	(7.90, 11.08)	<0.001 ***	12.57	(9.85, 16.03)	<0.001 ***	26.62	(21.52, 32.93)	<0.001 ***	27.76	(22.34, 34.51)	<0.001 ***

***p<0.001; **p<0.01; *p<0.05; ORs: odds ratios; CIs: confidence intervals

Supplementary Table 7 | ORs and 95% CIs for the comparison of access to supermarkets vs access to local food for food security concerns during the state of emergency (Model 3c-3e) and access to convenience stores vs access to local food for food security concerns in the future (Model 4c-4e)

	Food security concerns during the state of emergency									Food security concerns in the future								
	Model 3c			Model 3d			Model 3e			Model 4c			Model 4d			Model 24		
Variables	ORs	(95% CIs)	*P*	ORs	(95% CIs)	*P*	ORs	(95% CIs)	*P*	ORs	(95% CIs)	*P*	ORs	(95% CIs)	*P*	ORs	(95% CIs)	*P*
Allotment farm	1.90	(1.38, 2.64)	<0.001 ***							3.59	(2.58, 4.99)	<0.001 ***						
Home garden				1.31	(1.08, 1.58)	0.006**							1.37	(1.14, 1.64)	<0.001 ***			
Farm stand							1.64	(1.30, 2.05)	<0.001 ***							2.24	(1.78, 2.81)	<0.001 ***
Supermarket	1.48	(1.23, 1.78)	<0.001 ***	1.48	(1.23, 1.78)	<0.001 ***	1.48	(1.23, 1.78)	<0.001 ***									
Convenience store										1.39	(1.22, 1.59)	<0.001 ***	1.45	(1.26, 1.65)	0.001 ***	1.39	(1.22, 1.59)	<0.001 ***
Male	0.88	(0.76, 1.02)	0.10	0.91	(0.78, 1.08)	0.28	0.88	(0.76, 1.03)	0.12	1.00	(0.96, 1.05)	0.88	1.01	(0.93, 1.10)	0.74	1.01	(0.94, 1.08)	0.81
Older adult	1.91	(1.60, 2.27)	<0.001 ***	1.86	(1.55, 2.23)	<0.001 ***	1.92	(1.61, 2.29)	<0.001 ***	1.51	(1.28, 1.77)	<0.001 ***	1.49	(1.26, 1.76)	<0.001 ***	1.53	(1.30, 1.81)	<0.001 ***
Low-income household	0.79	(0.57, 1.08)	0.14	0.78	(0.58, 1.05)	0.10	0.78	(0.57, 1.06)	0.12	0.63	(0.48, 0.82)	0.001 ***	0.64	(0.49, 0.83)	0.001 ***	0.64	(0.49, 0.83)	0.001 ***
Living alone	0.89	(0.74, 1.08)	0.24	0.92	(0.76, 1.10)	0.35	0.91	(0.76, 1.10)	0.33	0.96	(0.82, 1.11)	0.58	0.94	(0.79, 1.11)	0.47	0.96	(0.82, 1.11)	0.57
Living with young child	1.00	(0.96, 1.04)	0.97	1.00	(0.96, 1.04)	0.98	1.00	(0.96, 1.04)	0.97	1.01	(0.94, 1.08)	0.84	1.01	(0.93, 1.10)	0.81	1.01	(0.94, 1.08)	0.84
Working from home	1.05	(0.90, 1.21)	0.54	1.09	(0.92, 1.30)	0.31	1.05	(0.90, 1.22)	0.52	1.17	(1.02, 1.35)	0.02*	1.22	(1.06, 1.41)	0.005 **	1.20	(1.05, 1.38)	0.001 **
Not working	1.02	(0.91, 1.15)	0.75	1.03	(0.89, 1.19)	0.67	1.02	(0.91, 1.14)	0.76	1.00	(0.95, 1.05)	0.97	1.00	(0.96, 1.04)	0.95	1.00	(0.95, 1.05)	0.95
Living in detached house	1.00	(0.97, 1.04)	0.92	1.00	(0.96, 1.04)	0.90	1.00	(0.95, 1.05)	0.87	1.14	(0.97, 1.33)	0.10	1.09	(0.93, 1.29)	0.27	1.10	(0.94, 1.29)	0.22
Living in area with farmland	1.00	(0.96, 1.04)	0.93	1.00	(0.97, 1.03)	0.93	1.00	(0.97, 1.03)	0.99	1.01	(0.94, 1.10)	0.74	1.02	(0.93, 1.12)	0.68	1.00	(0.96, 1.04)	0.91
1|2	0.03	(0.02, 0.04)	<0.001 ***	0.03	(0.02, 0.04)	<0.001 ***	0.03	(0.02, 0.04)	<0.001 ***	0.10	(0.08, 0.12)	<0.001 ***	0.10	(0.08, 0.12)	***	0.10	(0.08, 0.12)	<0.001 ***
2|3	0.18	(0.14, 0.23)	<0.001 ***	0.19	(0.15, 0.24)	<0.001 ***	0.19	(0.15, 0.23)	<0.001 ***	0.59	(0.51, 0.69)	<0.001 ***	0.61	(0.52, 0.71)	***	0.61	(0.52, 0.71)	<0.001 ***
3|4	0.93	(0.75, 1.16)	0.55	0.99	(0.79, 1.24)	0.92	0.96	(0.77, 1.19)	0.70	3.66	(3.12, 4.29)	<0.001 ***	3.70	(3.15, 4.34)	***	3.74	(3.19, 4.37)	<0.001 ***
4|5	12.43	(9.78, 15.80)	<0.001 ***	13.08	(10.20, 16.78)	<0.001 ***	12.76	(10.03, 16.23)	<0.001 ***	28.69	(23.15, 35.57)	<0.001 ***				28.97	(23.41, 35.84)	<0.001 ***

***p<0.001; **p<0.01; *p<0.05; ORs: odds ratios; CIs: confidence intervals

Supplementary Table 8 | ORs and 95% CIs for the association between sociodemographic status and access to local food: Allotment (Model 5b), home garden (Model 5b), and farm stand (Model 5c)

	Access to allotments Model 5a			Access to home gardens Model 5b			Access to farm stands Model 5c		
Variables	ORs	(95% CIs)	*P*	ORs	(95% CIs)	*P*	ORs	(95% CIs)	*P*
(Intercept)	0.02	(0.02, 0.04)	<0.001***	0.13	(0.10, 0.16)	<0.001***	0.06	(0.04, 0.08)	<0.001***
Male	1.07	(0.81, 1.42)	0.65	0.66	(0.53, 0.81)	<0.001***	1.02	(0.89, 1.18)	0.75
Older adult	1.17	(0.73, 1.87)	0.51	2.23	(1.73, 2.87)	<0.001***	0.99	(0.89, 1.11)	0.91
Low-income household	0.99	(0.81, 1.22)	0.96	1.00	(0.87, 1.16)	0.99	0.98	(0.77, 1.24)	0.86
Living alone	0.56	(0.33, 0.94)	0.03*	0.44	(0.32, 0.60)	<0.001***	0.62	(0.43, 0.89)	0.009**
Living with young child	1.53	(0.98, 2.39)	0.06	1.08	(0.84, 1.39)	0.56	1.26	(0.90, 1.75)	0.18
Working from home	3.02	(2.04, 4.49)	<0.001***	1.59	(1.25, 2.01)	<0.001***	1.81	(1.41, 2.32)	<0.001***
Not working	0.96	(0.70, 1.32)	0.79	1.04	(0.85, 1.27)	0.70	1.01	(0.89, 1.15)	0.89
Living in a detached house	1.19	(0.81, 1.75)	0.37	2.00	(1.62, 2.46)	<0.001***	1.43	(1.11, 1.84)	0.005**
Living in an area with farmland	1.00	(0.93, 1.08)	0.97	1.00	(0.94, 1.07)	0.95	1.65	(1.29, 2.11)	<0.001***

***p<0.001; **p<0.01; *p<0.05; ORs: odds ratios; CIs: confidence intervals

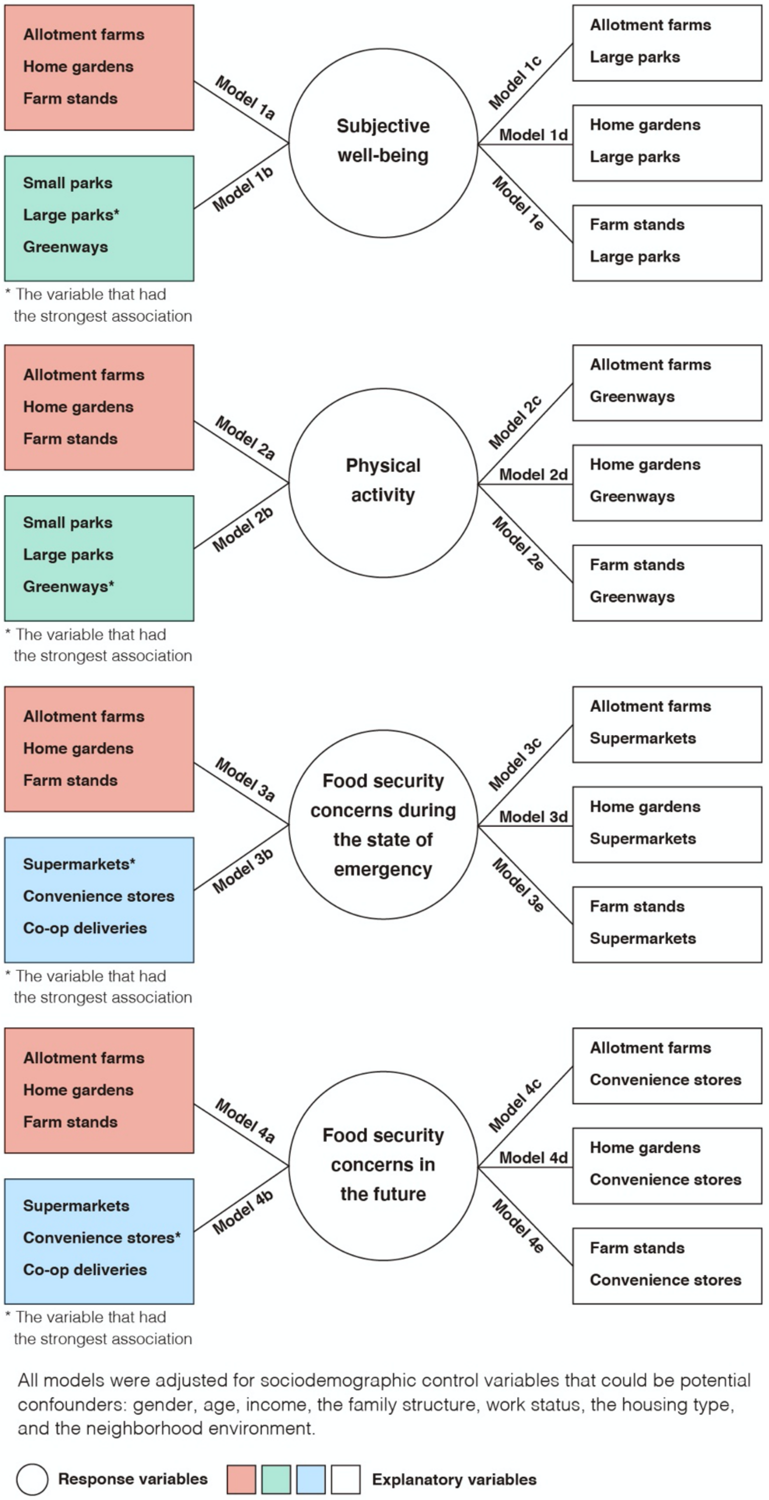

Supplementary Figure 1 | Structure of regression models

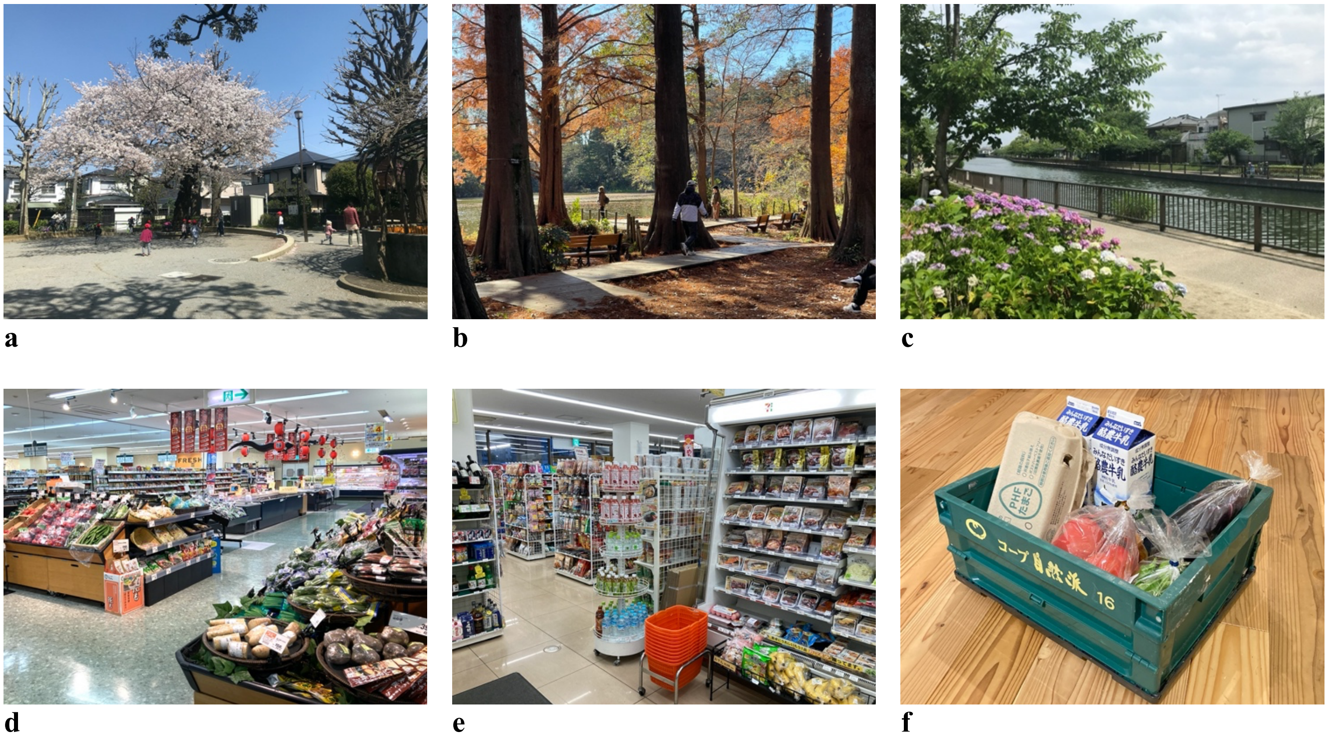

Supplementary Figure 2 | Other urban green spaces and other food purchasing sites

a-c, Other urban green spaces; small parks with some playground equipment and benches which are mainly for neighborhood residents living within walkable distance; (**a**), large parks with large open spaces of some size and sports facilities which are for all urban residents (**b**), and greenways including riverside paths (**c**). **d-e**, Other food purchasing sites; supermarkets (**d**), convenience stores (**e**), and co-op deliveries which are grocery delivery services managed by cooperative associations (**f**).

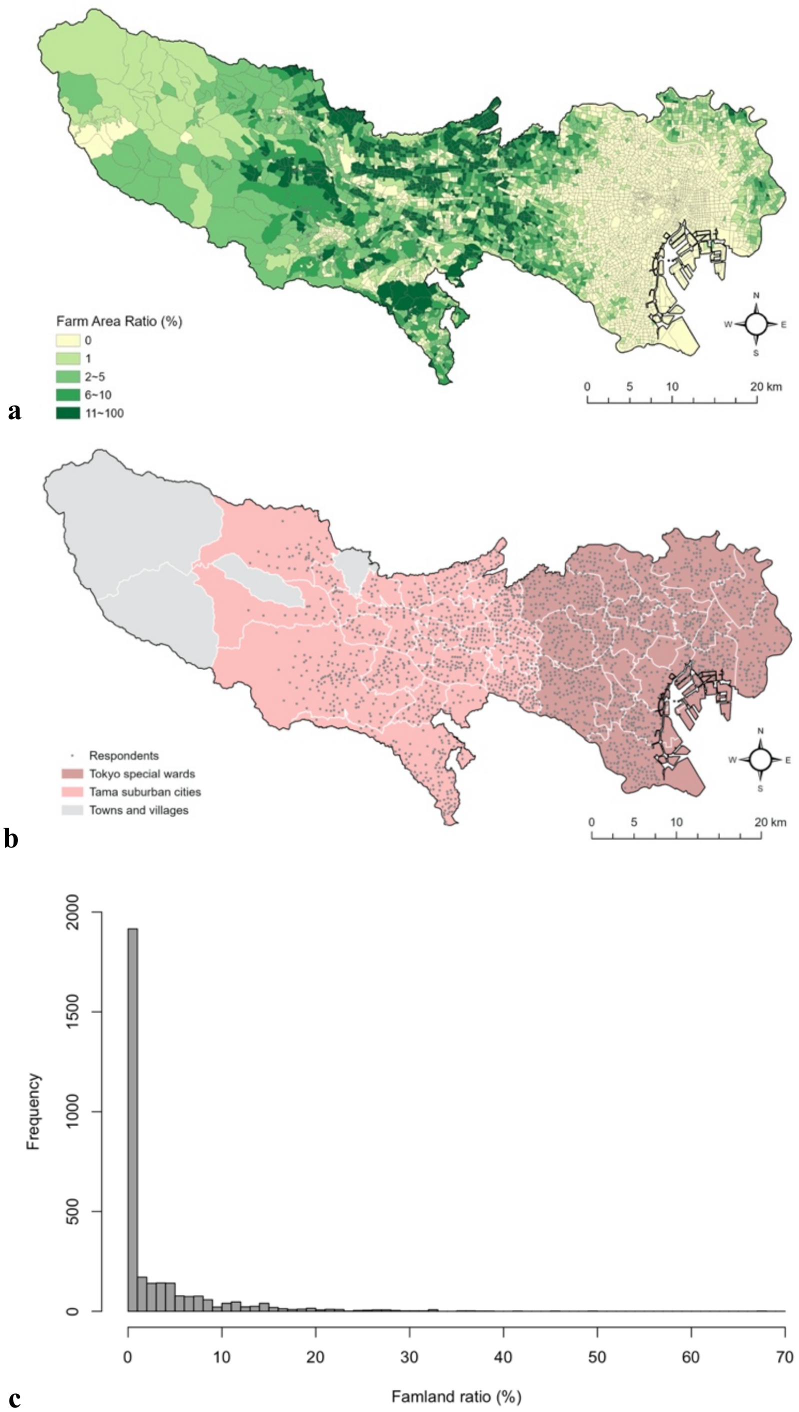

Supplementary Figure 3 | Spatial analysis of farmland in Tokyo

a, Farmland ratio by neighborhood called ‘*cho-cho-moku*’ in Tokyo. **b**, Location of the neighborhoods of respondents in the target area (i.e., Tokyo special wards and Tama suburban cities). **c**, Histogram of farmland ratio by the neighborhoods of respondents. The mean ratio is 3.3 (standard deviation 5.9).
