# Supplementary material for: Urban agriculture in walkable neighborhoods bore fruit for health and food system resilience during the COVID-19 pandemic
Source: NPJ Urban Sustain. 2023 Feb 1;3(1):4. doi: 10.1038/s42949-023-00083-3 (PMC9890428; doi:10.1038/s42949-023-00083-3)
Supplement: Supplementary file 2 — Reporting Summary [file 42949_2023_83_MOESM2_ESM.pdf]

nature portfolio

Corresponding author(s): Akiko Iida

Last updated by author(s): Dec 22, 2022

Reporting Summary

Nature Portfolio wishes to improve the reproducibility of the work that we publish. This form provides structure for consistency and transparency in reporting. For further information on Nature Portfolio policies, see our Editorial Policies and the Editorial Policy Checklist.

Statistics

For all statistical analyses, confirm that the following items are present in the figure legend, table legend, main text, or Methods section.

n/a	Confirmed	
☐	☒	The exact sample size (*n*) for each experimental group/condition, given as a discrete number and unit of measurement
☐	☒	A statement on whether measurements were taken from distinct samples or whether the same sample was measured repeatedly
☐	☒	The statistical test(s) used AND whether they are one- or two-sided<br>*Only common tests should be described solely by name; describe more complex techniques in the Methods section.*
☐	☒	A description of all covariates tested
☐	☒	A description of any assumptions or corrections, such as tests of normality and adjustment for multiple comparisons
☐	☒	A full description of the statistical parameters including central tendency (e.g. means) or other basic estimates (e.g. regression coefficient) AND variation (e.g. standard deviation) or associated estimates of uncertainty (e.g. confidence intervals)
☐	☒	For null hypothesis testing, the test statistic (e.g. *F*, *t*, *r*) with confidence intervals, effect sizes, degrees of freedom and *P* value noted<br>*Give P values as exact values whenever suitable.*
☒	☐	For Bayesian analysis, information on the choice of priors and Markov chain Monte Carlo settings
☒	☐	For hierarchical and complex designs, identification of the appropriate level for tests and full reporting of outcomes
☐	☒	Estimates of effect sizes (e.g. Cohen's *d*, Pearson's *r*), indicating how they were calculated

Our web collection on statistics for biologists contains articles on many of the points above.

Software and code

Policy information about availability of computer code

Data collection	No software was used in the data collection.
Data analysis	We used ArcGIS Pro 2.9 for spatial analysis and R version 4.0.2 for statistic analysis and plotting, along with the packages MASS, MuMln, and Tidyverse. The R code for our statistical analyses is available at https://github.com/AkikoII/UA-in-Tokyo-under-COVID-19/

For manuscripts utilizing custom algorithms or software that are central to the research but not yet described in published literature, software must be made available to editors and reviewers. We strongly encourage code deposition in a community repository (e.g. GitHub). See the Nature Portfolio guidelines for submitting code & software for further information.

Data

Policy information about availability of data

All manuscripts must include a data availability statement. This statement should provide the following information, where applicable:

- Accession codes, unique identifiers, or web links for publicly available datasets
- A description of any restrictions on data availability
- For clinical datasets or third party data, please ensure that the statement adheres to our policy

The study population data that were used for statistical analyses are not publicly available. Based on the ethical approval granted, only those registered as coresearchers can handle the data. To request access to the data, contact the corresponding author, A.I. The spatial data on farmlands (land use) and buildings in Tokyo that were used for geographic information system (GIS) analyses were provided by the Tokyo Metropolitan Government Basic Urban Planning Survey in

2016–2017. These data are not open data but can be used for research purposes (https://www.toshiseibi.metro.tokyo.lg.jp/seisaku/tochi_c/index.html). Other spatial data are publicly available. The data on the neighborhood division called "Cho-cho-moku" are available at e-Stat (e-stat.go.jp/gis), and the data on the municipality division and UPAs are available at the National Land Information Division (nlftp.mlit.go.jp/ksj).

Human research participants

Policy information about studies involving human research participants and Sex and Gender in Research.

Reporting on sex and gender	This study includes information on self-reporting gender. The gender information was included in the basic information of the online questionnaire platform provided by Macromill, Inc., an online research company. The company collected gender information by asking the following question. "Please choose Male or Female. If there is no applicable option, please select one of the two genders you would like to use this service as."
Population characteristics	See "Behabioural & social science study design"
Recruitment	An online questionnaire survey was conducted using the platform provided by Macromill, Inc. The questionnaire of this study was sent by email to those previously registered as monitors with the company. The company is one of the most largest companies that provide online questionnaire services in Japan. The number of monitors is approximately 11 million, which is 8.8 % of the Japanese population. Although online surveys have the potential for self-selection bias, we employed the online survey because it was the most feasible option during the COVID-19 emergency.
Ethics oversight	This study was approved by the Ethical Committee of the Graduate School of Engineering, The University of Tokyo (approval number: KE20-8).

Note that full information on the approval of the study protocol must also be provided in the manuscript.

Field-specific reporting

Please select the one below that is the best fit for your research. If you are not sure, read the appropriate sections before making your selection.

☐ Life sciences ☒ Behavioural & social sciences ☐ Ecological, evolutionary & environmental sciences

For a reference copy of the document with all sections, see nature.com/documents/nr-reporting-summary-flat.pdf

Behavioural & social sciences study design

All studies must disclose on these points even when the disclosure is negative.

Study description	We performed logistic regression analyses using a cross-sectional dataset of 3,135 adults in Tokyo to quantitatively verify the association of access to local food through urban agriculture (allotment farms, home gardens, farm stands) with subjective well-being, physical activity, and food security concerns of neighborhood communities in the context of the COVID-19 pandemic.
Research sample	A total of 4,126 adults living in Tokyo, aged 20 years and above, responded to the questionnaire in Japanese. Our sample population is 58% male and 23% older people, aged 65 years and above, while Tokyo's population is 49% male and 23% older people in 2020. Although the proportion of males is slightly higher, the sample is representative of the entire population.
Sampling strategy	We employed three-stage sampling method. First, the number of questionnaire respondents was adjusted in advance to include at least 1,000 people who were experienced in using allotment farms and/or farm stands. Among all the respondents, 1,030 (25%) were experienced, and 3,096 (75%) were not. Second, the number of respondents was adjusted during the recruitment process so that they would be evenly distributed across age groups (i.e., divided based on those in their 20s, 30s, 40s, 50s, 60s, and 70s and above) and places of residence (i.e., Tokyo special wards and Tama suburban cities).This was done to correct for age bias and living location bias in the monitored enrollees. After excluding the questionnaires of those with missing data (see "Data exclusions"), the analytical sample comprised3,135 participants. The sample size for allotment users was 147, that for home gardeners was 497, and that for farm stands was 326. Ten events per variable is widely advocated as the minimum standard for sample size consideration in logistic regression analysis. Since the number of variables used in this study was 12, the sample size should be at least 120. Therefore, our sampling meets the minimum criteria for sample size.
Data collection	An online questionnaire survey was conducted using the platform provided by Macromill, Inc., an online research company in Japan.
Timing	The survey was conducted between June 4 and 8, 2020, shortly after the end of the first declaration of a state of emergency by the Japanese National Government.
Data exclusions	After excluding the questionnaires of those with missing data (i.e., those who did not answer the income question, those who did not indicate their neighborhood, and those who did not properly complete the short version of the International Physical Activity Questionnaire), the analytical sample comprised3,135 participants.
Non-participation	All survey respondents consented to participate in Macromill's anonymous online survey. They understood the purpose of the survey

Non-participation	and were informed of their right to withdraw at any time.
Randomization	Participants were not allocated into experimental groups.

Reporting for specific materials, systems and methods

We require information from authors about some types of materials, experimental systems and methods used in many studies. Here, indicate whether each material, system or method listed is relevant to your study. If you are not sure if a list item applies to your research, read the appropriate section before selecting a response.

Materials & experimental systems

n/a	Involved in the study
☒	☐ Antibodies
☒	☐ Eukaryotic cell lines
☒	☐ Palaeontology and archaeology
☒	☐ Animals and other organisms
☒	☐ Clinical data
☒	☐ Dual use research of concern

Methods

n/a	Involved in the study
☒	☐ ChIP-seq
☒	☐ Flow cytometry
☒	☐ MRI-based neuroimaging
